# Development of a whole-cell biocatalyst for diisobutyl phthalate degradation by functional display of a carboxylesterase on the surface of *Escherichia coli*

**DOI:** 10.1186/s12934-020-01373-6

**Published:** 2020-05-29

**Authors:** Junmei Ding, Yang Zhou, Chaofan Wang, Zheng Peng, Yuelin Mu, Xianghua Tang, Zunxi Huang

**Affiliations:** 1 Engineering Research Center of Sustainable Development and Utilization of Biomass Energy, Ministry of Education, Yunnan Normal University, Kunming, 650500 Yunnan China; 2 Key Laboratory of Yunnan for Biomass Energy and Biotechnology of Environment, Kunming, 650500 Yunnan China; 3 Key Laboratory of Enzyme Engineering, Yunnan Normal University, Kunming, 650500 Yunnan China

**Keywords:** Cell surface display, Phthalic acid esters, Whole-cell biocatalyst, Diisobutyl phthalate, Carboxylesterase

## Abstract

**Background:**

Phthalic acid esters (PAEs) are widely used as plasticizers or additives during the industrial manufacturing of plastic products. PAEs have been detected in both aquatic and terrestrial environments due to their overuse. Exposure of PAEs results in human health concerns and environmental pollution. Diisobutyl phthalate is one of the main plasticizers in PAEs. Cell surface display of recombinant proteins has become a powerful tool for biotechnology applications. In this current study, a carboxylesterase was displayed on the surface of *Escherichia coli* cells, for use as whole-cell biocatalyst in diisobutyl phthalate biodegradation.

**Results:**

A carboxylesterase-encoding gene (*carEW*) identified from *Bacillus* sp. K91, was fused to the N-terminal of ice nucleation protein (*inpn*) anchor from *Pseudomonas syringae* and *gfp* gene, and the fused protein was then cloned into pET-28a(+) vector and was expressed in *Escherichia coli* BL21(DE3) cells. The surface localization of INPN-CarEW/or INPN-CarEW-GFP fusion protein was confirmed by SDS-PAGE, western blot, proteinase accessibility assay, and green fluorescence measurement. The catalytic activity of the constructed *E. coli* surface-displayed cells was determined. The cell-surface-displayed CarEW displayed optimal temperature of 45 °C and optimal pH of 9.0, using *p*-NPC_2_ as substrate. In addition, the whole cell biocatalyst retained ~ 100% and ~ 200% of its original activity per OD_600_ over a period of 23 days at 45 °C and one month at 4 °C, exhibiting the better stability than free CarEW. Furthermore, approximately 1.5 mg/ml of DiBP was degraded by 10 U of surface-displayed CarEW cells in 120 min.

**Conclusions:**

This work provides a promising strategy of cost-efficient biodegradation of diisobutyl phthalate for environmental bioremediation by displaying CarEW on the surface of *E. coli* cells. This approach might also provide a reference in treatment of other different kinds of environmental pollutants by displaying the enzyme of interest on the cell surface of a harmless microorganism.

## Background

Phthalic acid esters (PAEs) are a class of organic compounds that are widely used as plasticizers or additives during the industrial manufacturing of plastic products [1]. PAEs provide flexibility, durability, and elasticity to polyvinyl chloride (PVC) resins and other polymers by physically interacting with polymeric matrices which make PAEs directly and/or indirectly migrating into the environment during industrial production, utilization, or disposal. Consequently, PAEs are detected not only in aquatic but also in terrestrial environments [2]. However, after over a half century of supposedly safe utilization, several experimental studies have demonstrated that PAEs could cause adverse effects to human health [3], including disrupt the endocrine systems [4], induce reproductive toxicity [5] and hepatocellular tumors, harm fetal health [3, 6], and so on. As one of the main plasticizers in PAEs, diisobutyl phthalate (DiBP) has permanently been banned by the U.S. Consumer Products Safety Commission (CPSC) because of its reproductive toxicity [7]. Therefore, the perception of environmental and health risks imposed by PAEs has fundamentally changed and important issues pertaining to the environmental fate of PAEs have been raised.

As sustainable and hazardous contamination of PAEs to environment and our human health, biodegradation of PAEs in the environment attracted more attention [1, 2, 6]. Microbial degradation especially bacteria-mediated biodegradation is considered the most promising method for removing PAEs from polluted environments. Several microorganisms and their related critical enzymes capable of degrading PAEs were summarized in Table 1, bacteria from genus of *Sphingobium* [8], *Pseudomonas* [9], *Bacillus* [10], *Sulfobacillus* [11], *Acinetobacter* [12], *Rhodococcus* [13], *Fusarium* [14, 15], *Gordonia* [16], and *Micrococcus* [17] were included. Moreover, esterases from tissues [18] or uncultured microorganisms [19] with PAEs biodegradation ability were also reported. Based on the identification of associated metabolic intermediates, two steps are involved in the metabolic pathways associated with PAE biodegradation: (i) transformation of PAEs to phthalic acid (PTH) and (ii) complete degradation of PTH. Esterases/hydrolases expressed by microorganisms played critical role in both steps [20]. However, until now, only few esterases/hydrolases involved in PAE decomposition have been characterized.

**Table 1:** Bacteria and related esterases involved in PAEs biodegradation

Esterases	Sources	Substrates	References
Carboxylesterase	*Sphingobium yanoikuyae*	Dinbutyl phthalate	[8]
Hydrolase	*Pseudomonas mosselii*	Mono-2-ethylhexyl phthalate	[9]
CarEW	*Bacillus* sp. K91	Diisobutyl phthalate	[10]
EstS1	*Sulfobacillus acidophilus*	Phthalate esters	[11]
PE hydrolase	*Acinetobacter* sp. M673	Dibutyl phthalate	[12]
PatE	*Rhodococcus jostii* RHA1	Monoalkyl phthalate	[13]
DMT esterase	*Fusarium* sp. DMT-5-3	Dimethyl terephthalate	[14]
Cutinase	*Fusarium oxysporum*	Phthalate esters	[15]
Esterase	*Gordonia* sp. P8219	Mono-2-ethylhexyl Phthalate	[16]
Esterases	*Micrococcus* sp. YGJ1	Monoalkyl phthalates	[17]
Esterase	Pancreatic cholesterol	Phthalate esters	[18]
DphB	Metagenomics library	Dibutyl phthalate	[19]

Carboxylesterases (EC 3.1.1.1), also known as esterases, are widely distributed in nature and play multiple important functions in the detoxification of various harmful exogenous compounds, such as herbicides [21], pesticides [22], and so on. With a catalytic triad composed of Ser-Asp (or Glu)-His and a consensus sequence (G-X-S-X-G) around the active site serine residues, carboxylesterases belong to the α/β hydrolase superfamily and catalyze the hydrolysis of carboxylic ester bonds (< 10 carbon atoms). Together with lipases, both are very important industrial enzymes and are widely distributed in nature [23]. Carboxylesterases or lipases exhibit stable thermostability, accept wide range of substrates, require no cofactor, maintain high regio/stereo-specificity, remain stable in organic solvents. These properties make carboxylesterases or lipases to be used as biocatalysts in a variety of industrial processes, including biochemical, food, pharmaceutical, and biological purposes [24]. However, the purification costs, low catalytic activities and poor enzyme stability of the requisite enzymes are all concerns for large scale practical applications.

Many of these problems mentioned above can be solved by displaying useful foreign enzymes on live microbial cell surface by fusing them with appropriate anchoring motifs. Anchorage of target enzymes on the outer membrane of model microorganisms allows direct enzymatic reaction with substrates with no need of crossing the membrane barrier and purifying the enzymes which significantly reduce the cost of whole cell biocatalyst preparation and application [25]. Previous investigations showed that the microbial surface display systems have been successfully applied in various fields, including food industry [26], bioremediation [27], biofuel [28], biological synthesis [29], and so on. Among the anchoring motifs, the truncated N-terminal domain of ice nucleation protein (INP) identified from *Pseudomonas syringae* has been proven to be an efficient carrier [25]. However, the INP-mediated surface display method has not been used till now for the PAEs biodegradation although various successful applications of INP-anchored functional proteins have been reported.

In this study, a carboxylesterase, CarEW, was identified from *Bacillus* sp. K91 and functionally displayed on the surface of *E. coli* cells by fusing CarEW with the INPN anchoring motif. The environmentally friendly engineered *E. coli* strain was endowed with the capacity to degrade PAEs and could be potentially used for further environmental bioremediation. Additionally, this study may also provide a method for the biodegradation of other environmental pollutants.

## Results

### Expression of CarEW/GFP and INPN/CarEW/GFP fusion proteins

**Fig. 1:**
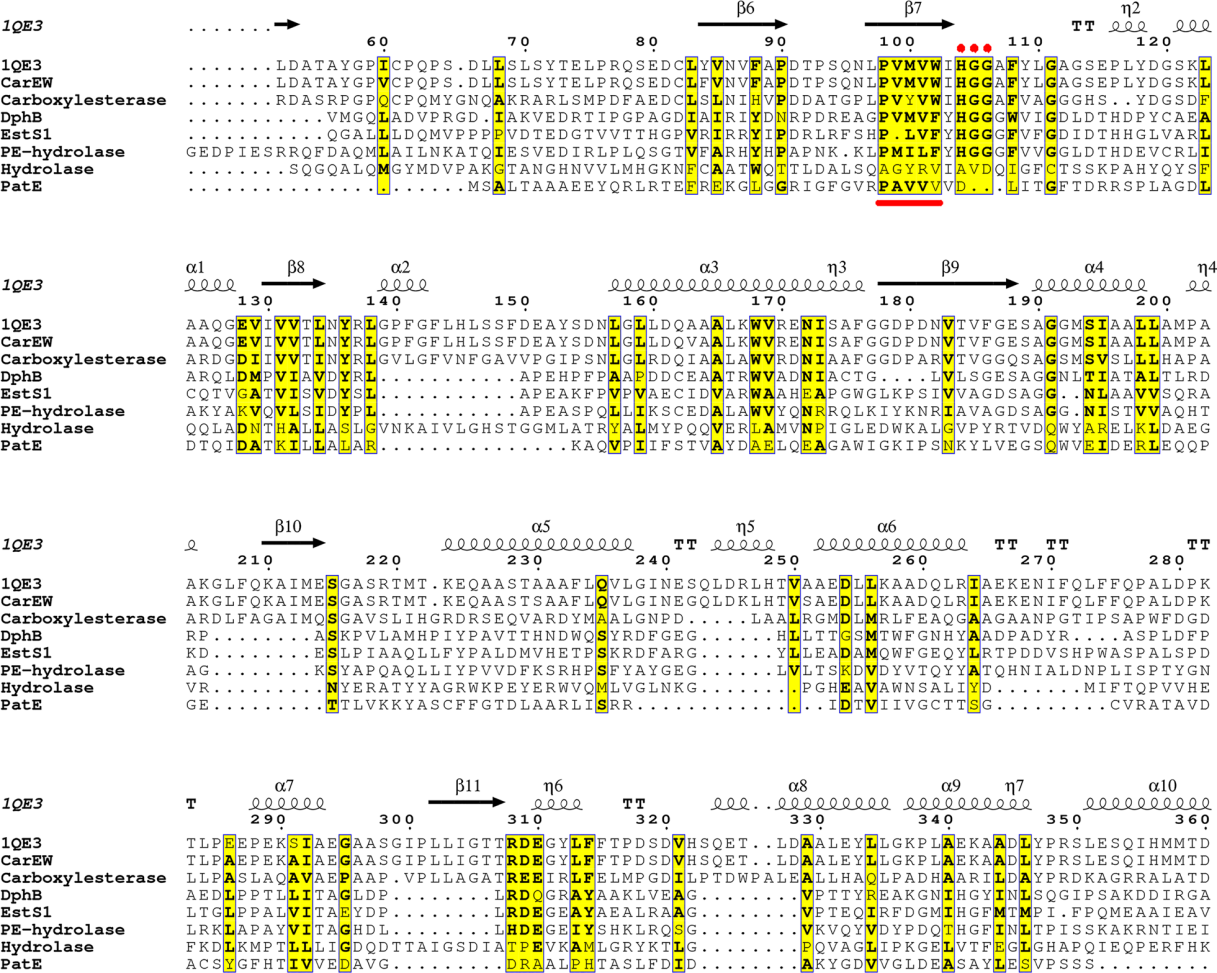
Multiple sequence alignment between CarEW and some previously reported esterases with PAEs biodegradation capacities. Sequences retrieved from the NCBI database and were aligned by CLUSTAL W and were rendered using ESPript output. Sequences are grouped according to similarity. Esterase with a known three-dimensional structure (PDB: 1QE3) from *Bacillus subtilis*; KMW28714.1, Carboxylesterase from *Sphingobium yanoikuyae*; AGY55960.1, DphB from metagenomics library; AEW03609.1, EstS1 from *Sulfobacillus acidophilus* DSM 10332; AFK31309.1, PE-hydrolase from *Acinetobacter* sp. M673; WP_023629646.1, alpha/beta hydrolase from *Pseudomonas mosselii*; ABH00399.1, PatE from *Rhodococcus jostii* RHA1. Conserved amino acids are highlighted in a yellow font on a white background. The analysis revealed the presence of tripeptide HGG (red dots on top of the sequences) and PVMVW (underline in red) in most of test strains. Symbols above sequences represent the secondary structure, springs represent helices, and arrows represent β-strands

The CarEW encoding gene *carEW* with a 1464-long ORF was amplified from *Bacillus* sp. K91 and the recombinant *E. coli* strain BL21(DE3)-pEASY-E2/*carEW* was constructed previously in our lab [10]. CarEW was composed of 487 amino acids and had a molecular mass of approximately 53.76 kDa with a pI of 4.88. Sequence alignment showed that CarEW shares less than 37% sequence similarity with some reported esterases which were capable of degrading PAEs (Fig. 1).

**Fig. 2:**
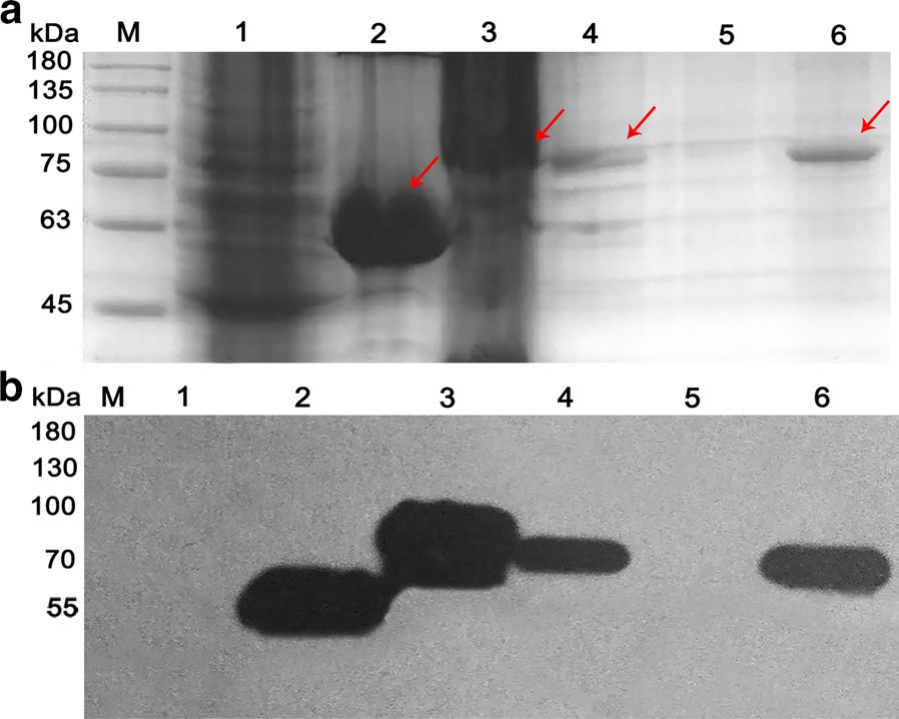
Expression of recombinant fusion proteins: CarEW, CarEW-GFP and INPN-CarEW-GFP. (a) SDS-PAGE and (b) western blot of lysates of *E. coli* harboring pET-28a(+) series plasmids. Lane M, protein marker; Lane 1, *E. coli* cells harboring pET-28a(+); Lane 2, *E. coli* cells harboring pET-28a(+)/*carEW*; Lane 3, *E. coli* cells harboring pET-28a(+)/*carEW*/*gfp*; Lane 4, 5, 6, cytoplasmic fraction, inner membrane, and outer membrane of *E. coli* cells harboring pET-28a(+)/*inpn*/*carEW*/*gfp*, respectively. Anti-His monoclonal antibody was used as a 1:1000 dilution

In this study, recombinant plasmids pET-28a(+)/*carEW*, pET-28a(+)/*carEW*/*gfp*, and pET-28a(+)/*inpn*/*carEW*/*gfp* were constructed and transformed into *E. coli* BL21(DE3) strain for the expression of CarEW, CarEW-GFP, and INPN-CarEW-GFP fusion proteins during the growth phase, respectively. *E. coli* BL21(DE3) strain containing the blank vector pET-28a(+) was used as an experimental control. Expression patterns of the fusion proteins were detected by sodium dodecyl sulfate polyacrylamide gel electrophoresis (SDS-PAGE). The results showed that a band corresponding to CarEW at ~ 53 kDa and ~ 80 kDa (CarEW, ~ 53 kDa; GFP, ~ 27 kDa; CarEW-GFP, ~ 80 kDa) appeared in *E. coli* BL21(DE3) cells harboring pET-28a(+)/*carEW* (Fig. 2a, lane 2, indicated by arrow) and pET-28a(+)/*carEW/gfp* (Fig. 2a, lane 3, indicated by arrow), respectively. For *E. coli* BL21(DE3) cells harboring pET-28a(+)/*inpn*/*carEW*/*gfp*, a band ~ 80 kDa appeared separately in the cytoplasmic fraction (Fig. 2a, lane 4, indicated by arrow) and outer membrane fraction (Fig. 2a, lane 6, indicated by arrow), while no band was detected in the inner membrane fraction (Fig. 2a, lane 5). No band was detected in the control, *E. coli* BL21(DE3) cells harboring the blank vector pET-28a(+) (Fig. 2a, Lane 1). These results indicated that the synthesized fusion proteins had been expressed correctly in different cell fractions and the size of the corresponding proteins matched well with the calculated molecular masses.

For the recombinant plasmids, pET-28a(+)/*carEW*, pET-28a(+)/*carEW*/*gfp*, and pET-28a(+)/*inpn*/*carEW*/*gfp*, all recombinant proteins were fused with 6× His tag at the C-terminal of the fusion proteins. The western blot analysis of the expressed proteins using anti-His monoclonal antibody further revealed clear signs of all corresponding proteins from subcellular fractions of *E. coli* BL21(DE3) containing pET-28a(+)/*carEW* (Fig. 2b, lane 2, CarEW, ~ 53 kDa), *E. coli* BL21(DE3) containing pET-28a(+)/*carEW*/*gfp* (Fig. 2b, lane 3, CarEW-GFP, ~ 80 kDa), and *E. coli* BL21(DE3) containing pET-28a(+)/*inpn*/*carEW*/*gfp* (Fig. 2b, lane 4 and 6, CarEW-GFP, ~ 80 kDa), whereas none was detected in the control (Fig. 2b, lane 1).

### Surface localization analysis of INPN-CarEW-GFP fusion protein on *E. coli* cells

Proteinases cannot cross the outer membrane of the cell, and, therefore, only surface-displayed proteins can be degraded by proteinases [30]. Therefore, evidence for the localization of target proteins on the cell surface can be proved by a proteinase accessibility assay. Proteinase K is a subtilisin-related serine proteinase that exhibits broad substrate specificity and hydrolyzes a variety of peptide bonds. After treatment with proteinase K for 1 h, the CarEW activity of *E. coli* BL21(DE3) cells carrying pET-28a(+)/*inpn*/*carEW* (OD_600_ = 1.0) decreased approximately 50% indicating that the surface-displayed CarEW was degraded by proteinase K, whereas, no obvious activity reduction was observed for *E. coli* BL21(DE3) cells carrying pET-28a(+)/*carEW* (OD_600_ = 1.0). This result supported the profile of western blot above that CarEW was approximately half localized on the surface, and half was distributed in the cytoplasmic for the *E. coli* BL21(DE3) cells carrying pET-28a(+)/*inpn*/*carEW* plasmid.

**Fig. 3:**
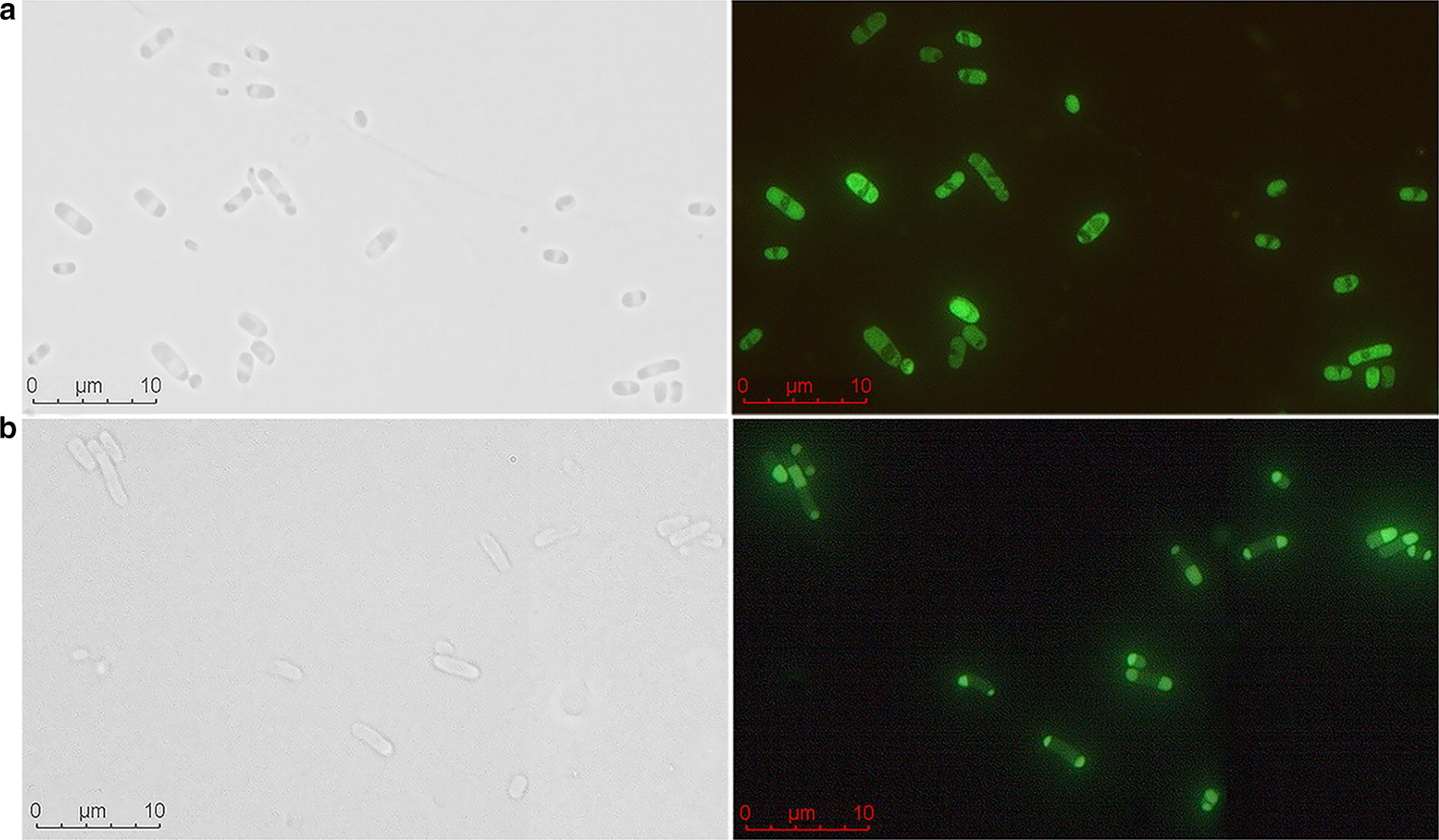
Fluorescence micrographs of recombinant *E. coli* BL21 (DE3) strain. **a***E. coli* BL21(DE3) cells carrying pET-28a(+)/*carEW/gfp* and **b** pET-28a(+)/*inpn/carEW/gfp*, respectively. Left panel, microphotographs were taken under visible light; Right panel, fluorescence microphotographs

To further confirm the presence of INPN-CarEW-GFP fusion protein on the cell surface, green fluorescence was observed. Under fluorescence microscopy, green fluorescence was concentrated at both poles or on membrane for cells containing plasmid pET-28a(+)/*inpn*/*carEW*/*gfp* (Fig. 3b, right panel). However, in the control cells, *E. coli* BL21(DE3) carrying pET-28a(+)/*carEW*/*gfp* without INPN anchor protein, the green fluorescence distributed evenly for the whole cells (Fig. 3a, right panel). So, the result suggested that INPN-CarEW-GFP fusion was correctly displayed on the surface of *E. coli* BL21(DE3) cells.

### Enzymatic activity and stability of *E. coli* BL21(DE3) strain expressing INPN-CarEW fusion protein

**Fig. 4:**
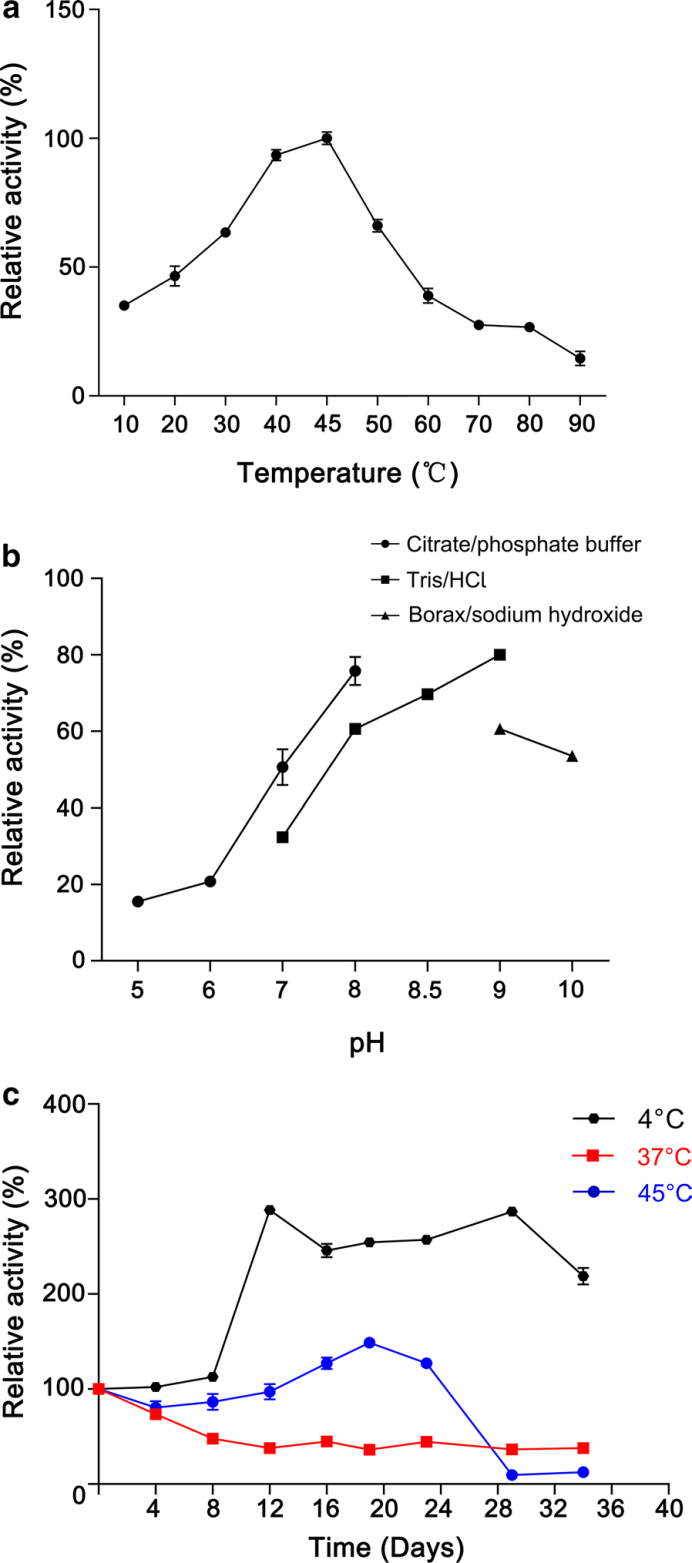
**a** The optimum temperature, **b** pH, and **c** Long-term stability of the whole cell biocatalyst. Residual activities were determined periodically for over a month. Values are the means of three replicates ± the standard deviation

Enzymatic activity of CarEW was determined as reported previously [10]. The substrate specificity of *E. coli* BL21(DE3) cells displaying CarEW was determined using the same concentrations of various *p*-NP substrates. The engineered *E. coli* cells were active on *p*-NPC_2_ to *p*-NPC_8_ and displayed maximal enzymatic activity toward *p*-NPC_2_ (Additional File 1: Figure S1). The optimal temperature and pH of the whole cell biocatalyst were investigated. As shown in Fig. 4a, the enzymatic activity increased linearly from 10 °C to 45 °C and the maximal activity was detected at 45 °C. More than 40% of the enzyme activity was observed between 20 and 55 °C, and the whole cell biocatalyst exhibited more than 20% of the original enzymatic activity when the temperature reached 80 °C. The optimal pH for the whole cell biocatalyst was determined at pH 9.0 and more than 40% of enzymatic activity was kept at pH values ranging from pH 6.5 to 9.0. In addition, more than 40% of maximum activity was observed at pH 10.0 (Fig. 4b). Under both optimal conditions, the enzymatic activity of the whole cell biocatalysts was demonstrated to be a *K*_*cat*_ of 26.46 ± 0.76 s^−1^ and *K*_*cat*_*/K*_*m*_ of 833.23 s^−1^ mM^−1^ (U/per OD_600_), respectively.

For surface expression approach, two concerns should be taken into consideration, one is the growth inhibition of cell, and the other is the stability of whole cell biocatalysts. To determine whether the surface display of INPN-CarEW fusion inhibits growth of the cell, growth profile of *E. coli* BL21(DE3) strain carrying pET-28a(+)/*carEW* or pET-28a(+)/*inpn*/*carEW* were compared. Two strain reached almost the same final density after incubated for 2 days, and no growth inhibition was observed for cells expressing INPN-CarEW fusion protein. To investigate the stability, whole cell enzymatic activity of the suspended cultures was determined periodically. The equal volume of engineered whole cell biocatalysts that suspended in citrate-Na_2_HO_4_ buffer (50 mM, pH 9.0) at 4 °C, 37 °C, and 45 °C, respectively, and residual activity of CarEW was determined intermittently for more than 1 month. No activity decrease of the whole cell biocatalyst was observed, and ~ 200% of the original enzymatic activity was detected when incubated at 4 °C for more than 1 month. When the temperature reached 45 °C, the enzymatic activity of engineered cells remained at essentially the original level up until 24th day. Soon after, 90% of CarEW activity was lost and the activity subsequently fluctuated at around 10% of the original activity level for the last 10 days. At 37 °C, the *E. coli* BL21(DE3) strain that surface expressed INPN-CarEW fusion protein exhibited ~ 50% of the original activity over 1 month (Fig. 4c). These results illustrated that the surface-displayed INPN-CarEW fusion neither inhibited cell growth nor caused instability of the cell.

### Degradation efficiency of DiBP by *E. coli* BL21(DE3) strain expressing INPN-CarEW fusion protein

**Fig. 5:**
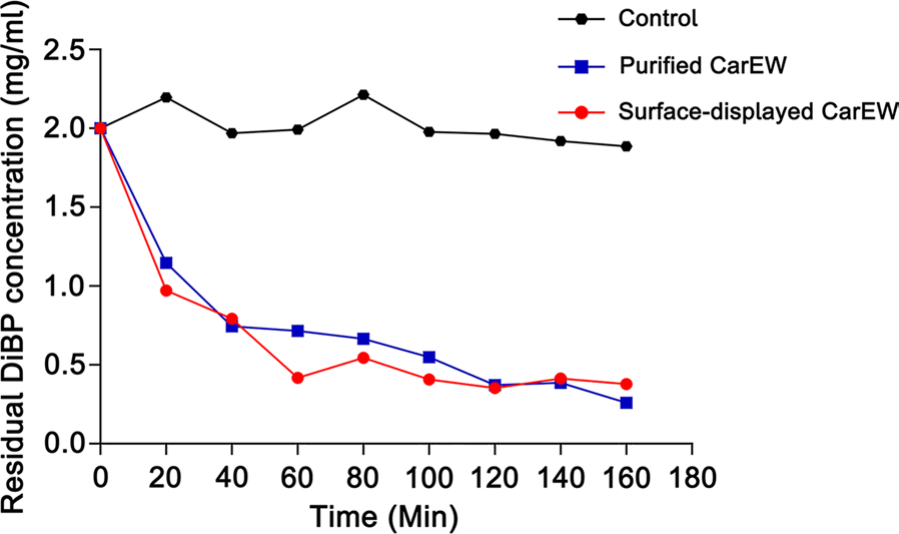
DiBP degradation by purified CarEW and the whole cell biocatalyst. The error bars represent the mean ± SD (*n *= 3)

In order to test the degradation efficiency of DiBP by *E. coli* BL21(DE3) strain expressing INPN-CarEW fusion protein, 10 U of whole cell biocatalyst and 10 U of purified CarEW was incubated with 2 mg/ml DiBP at 45 °C, respectively. No purified enzyme or whole cell biocatalyst was added in the control. As shown in Fig. 5, ~ 1.5 mg/ml DiBP was hydrolyzed in 120 min by both biocatalysts, and the biodegradation rate of CarEW surface-displayed cells is a little faster than that of the purified CarEW at 40 min to 120 min. Therefore, the biodegradation of DiBP was comparable between the whole cell biocatalyst and purified CarEW. However, it is more attractive for the whole cell biocatalyst to be applied in practical use because there are many advantages for the whole cell biocatalyst, such as low cost to obtain, stability and so on.

## Discussion

With the fast development of sequencing technologies, a large number of cultured and uncultured microorganisms were sequenced and their functional genes were annotated. *Bacillus* sp. K91, a thermophilic bacterium which can grow from 50 to 70 °C, was isolated by our lab from a hot spring water in Teng Chong, Yunnan Province, China. Based on the genome sequencing and annotation, a carboxylesterase, CarEW was cloned and expressed in *E. coli* BL21(DE3) [10]. Over the past few years, the heterologous proteins have been successfully displayed on the surface of bacteria or fungi, exhibiting promising prospects in many biotechnological processes [25–29].

In the present study, CarEW was displayed on the surface of *E. coli* BL21(DE3) using an ice nucleation protein anchor. Using this approach, CarEW on the bacterial surface could be produced and anchored simultaneously. Moreover, this whole cell biocatalyst can be stored and re-used easily, which can be directly used after cultivation and harvest. As shown by the SDS-PAGE (Fig. 2a), western blot (Fig. 2b) and proteinase K accessibility assay results, approximately 50% of total CarEW was displayed on the *E. coli* BL21(DE3) surface, and ~ 50% of CarEW was expressed in the cytoplasm. Consistent with several other previously reports, less than 50% target proteins were expressed on the surface of *E. coli* or in other surface-displaying bacteria, *Pseudomonas putida*, for example [31, 32]. Surface display systems mediated by the full length or truncated INP anchors from *P. syringae* have been extensively exploited in *E. coli*, *Pseudomonas* sp., and other species. In the future, other anchors, such as outer membrane protein A (OmpA), OmpC, or OmpF, and so on, and other surface-displayed microorganisms, *Saccharomyces cerevisiae*, for example, are deserved to be determined [33, 34]. In addition, the green fluorescence was concentrated at both poles or on outer membrane of *E. coli* BL21(DE3) strain carrying pET-28a(+)/*inpn/carEW/gfp* plasmid, while GFP distributed evenly for *E. coli* BL21(DE3) strain carrying pET-28a(+)/*carEW/gfp* plasmid, without the INP anchor motif (Fig. 3). This result observed here is consistent with other previous reports using the same INP-mediated system [35]. As mentioned above, about 50% of target proteins can be displayed on the surface of cells when used the INP-mediated surface-displayed system, as also observed by CarEW. Therefore, we supposed that the GFP might be buried in the cell wall and not successfully displayed on the surface, then lead to the GFP signal was concentrated at both pores for cells carrying pET-28a(+)/*inpn/carEW/gfp* plasmid. These might also explain that the activity of whole cell biocatalyst increased at the 12th day might be due to the release of CarEW resided in the cytoplasm caused by cell lysis, which was similar to an organophosphorus hydrolase using the same surface display system [27].

**Fig. 6:**
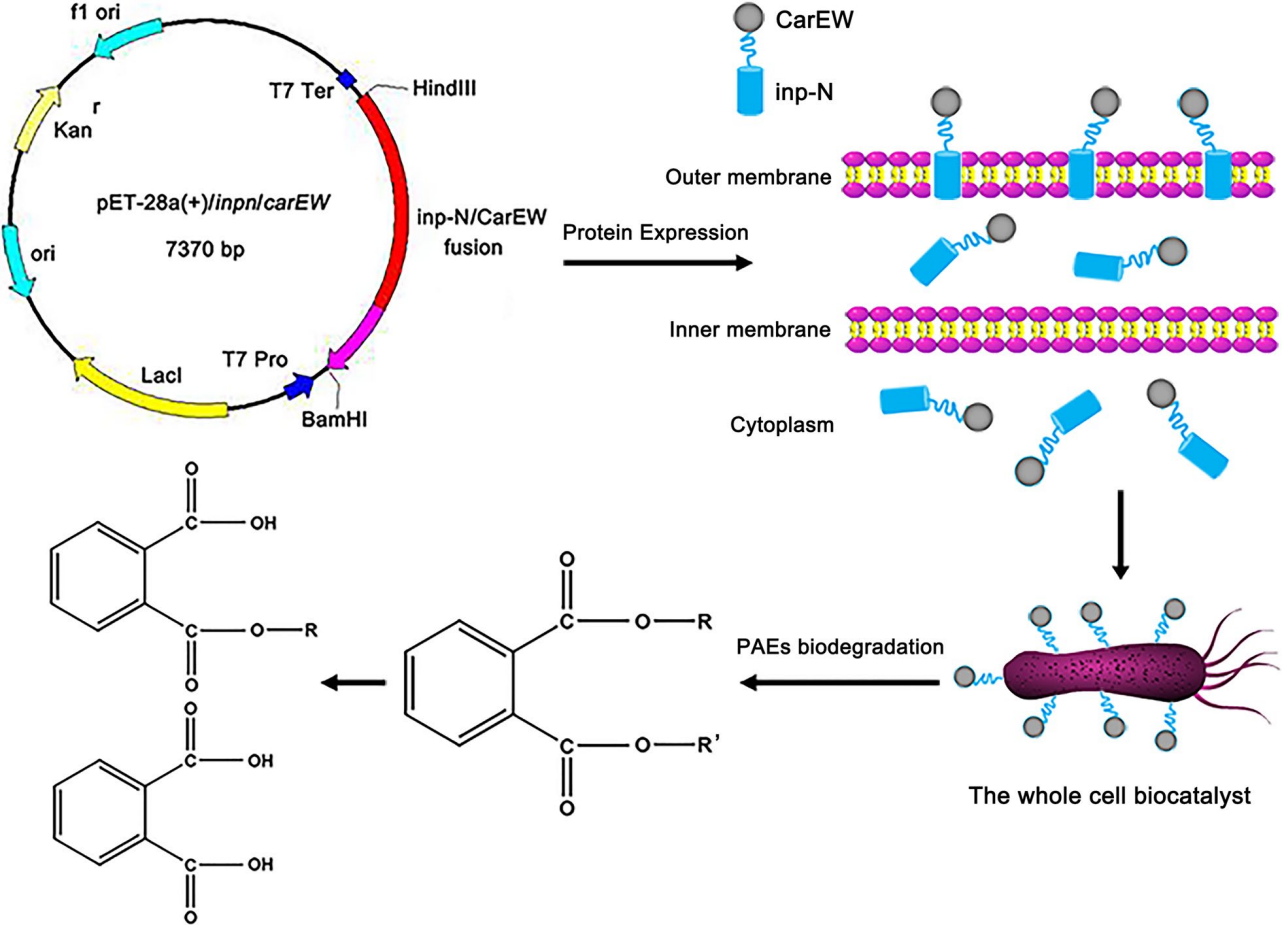
The schematic diagram for CarEW surface display engineered strain construction and its applications for PAEs biodegradation

The use of engineered microorganisms as bioremediating biocatalysts to eliminate pollutants in the environment represents a promising strategy [27, 31]. In this present study, a laboratory-scale whole cell biocatalyst used for DiBP degradation was developed, and a schematic diagram represented the progress was constructed (Fig. 6). To the best of our knowledge, this work is the first approach to degrade hazardous DiBP using engineered bacterial cells with surface-displayed carboxylesterase. In the natural environment, many factors influence the biodegradation efficiencies, such as the fluctuating environmental conditions, different microbial populations, complex contaminants, and so on. Therefore, although the biodegradation efficiency of DiBP is comparable between the purified CarEW and CarEW whole cell biocatalyst, the stability (stable at 4 °C and 45 °C) (Fig. 4c) and availability made the whole cell biocatalyst better suitable for practical environmental bioremediation. Additionally, there are usually a variety of contaminants exist at a one single site in most situations, thus, the application of this CarEW surface-displayed engineered strain for removal of PAEs besides DiBP need to be further investigated.

## Conclusions

Here, a surface displayed system based on INPN as a carrier protein for DiBP biodegradation was developed. The INPN-CarEW surface display fusion protein had no negative effect on cell growth or membrane integrity. This engineered strain had the capacity to degrade DiBP, which emphasizes high potential to use this strain for removal of other kinds of PAEs pollutants in the environment or use this strategy to develop other bioremediation approaches.

## Methods

### Substrates, reagents and plasmids

*p*-NP esters with various carbon chain lengths (*p*-NPC_2_ to *p*-NPC_16_) were purchased from Sigma-Aldrich (USA) or TCI (Tokyo, Japan). Diisobutyl phthalate (DiBP), dibutyl phthalate (DBP), Bis (2-ethylhexyl) phthalate (DEHP), and diethyl phthalate (DEP) were purchased from J&K Scientific Ltd., China. pEASY-E2 expression kit and fast *pfu* DNA polymerase were provided by TransGen Biotech (Beijing, China). *Escherichia coli* BL21(DE3) and pET-28a(+) expression vector was from Novagen (USA). Qiagen provided the Nickel-NTA agarose (Germany). pEGFP-N3, pMD18-T and restriction enzymes *Bam*HI and *Hind*III were purchased from Takara. Luria–Bertani (LB) bacteria growth medium was obtained from Thermo Fisher Scientific (USA). All other chemicals were at least analytical grade and were obtained from Sigma (USA) or Sinopharm Chemical Reagent (Shanghai, China).

### Plasmid construction

The *inak* gene was biosynthesized by Sangon Biotech, Shanghai, China, and subcloned into pMD18-T to generate pMD18-T/*inak*. To construct pET-28a(+)/*carEW*, *carEW* gene was amplified using primers P1 and P2 from a previously constructed plasmid, pEASY-E2/*carEW* [10]. The PCR products were digested with *Bam*HI and *Hin*dIII, and ligated with similarly digested pET-28a(+) to generate pET-28a(+)/*carEW*. To construct pET-28a(+)/*carEW/gfp*, *gfp* gene was amplified with primers P3 and P4 using pEGFP-N3 as a template, and ligated to similarly *Hin*dIII digested pET-28a(+)/*carEW*. For the construction of pET-28a(+)/*inpn/carEW*, the N-terminal domain of *inak* gene (named as *inpn* gene) was amplified from plasmid pMD18-T/*inak* with primers P5 and P6, and then was ligated to similarly *Bam*HI digested pET-28a(+)/*carEW.* To construct pET-28a(+)/*inpn/carEW/gfp*, *gfp* gene was amplified with primers P7 and P8, and the PCR products were ligated to similarly *Bam*HI digested pET-28a(+)/*inpn/carEW*. All the constructed recombinant plasmids were confirmed by sequencing (Sangon Biotech, Shanghai, China). A schematic diagram for the construction of recombinant plasmids are shown in Additional file 2: Figure S2. Primers and plasmids used in this study are summarized in Additional file 3: Table S1.

### Enzyme activity of surface-displayed CarEW

Esterase activity was determined at 405 nm by measuring the absorbance of liberated *p*-NP as reported previously [10]. The amount of enzyme required to release 1 μM *p*-NP per minute was defined as one unit of enzyme activity (U). Different buffers (50 mM): citrate/phosphate buffer (pH 5.0–8.0), Tris/HCl (pH 8.0–9.0), and boric acid/borax (pH 9.0–10.0) were used to determine the optimal pH of surface-displayed CarEW. Temperatures ranging from 0 to 80 °C was used to determine optimal temperature at pH 9.0 using *p*-NPC_2_ as substrate. Substrate specificity were investigated using different *p*-NP esters (*p*-NPC_2_ to *p*-NPC_16_). Kinetic was determined using different concentrations of *p*-NPC_2_ (0.12 to 1.2 mM) at 45 °C and pH 9.0. The Michaelis–Menten constant (*K*_*m*_) and maximum velocity (*V*_*max*_) were investigated by a nonlinear regression method.

To determine the stability of whole cell biocatalyst, *E. coli* BL21 cells harboring pET-28a(+)/*inpn*/*carEW* were resuspended in 50 mM Tris/HCl buffer (pH 9.0) after induction and incubated at 4 °C, 37 °C, and 45 °C, respectively. An identical volume of sample solution was extracted at regular intervals for over a month to facilitate CarEW activity determination.

### Cell fractionation, western blot and proteinase K accessibility assay

The pET-28a(+) series plasmids including pET-28a(+), pET-28a(+)/*carEW*, pET-28a(+)/*inpn*/*carEW*, pET-28a(+)/*carEW*/*gfp*, and pET-28a(+)/*inpn*/*carEW*/*gfp* were transformed into *E. coli* BL21(DE3) cells and were cultivated in LB medium supplemented with 100 μg/ml ampicillin at 37 °C, separately. 0.1 mM of Isopropyl β-D-1-thiogalactopyranoside (IPTG) was added when OD_600_ reached 0.5, respectively. Then, the cell cultures were maintained at 20 °C for another 20 h for protein induction. After harvested (12,000×*g* for 30 min, 4 °C) and resuspended in PBS buffer (pH 7.0), cell pellets were disrupted by sonication (7 s, 150 w) for 15 min on ice, and the supernatant was collected (12,000×*g* for 30 min, 4 °C). Total membrane fractions were obtained by centrifugation at 37,000 rpm for 4 h (at 4 °C). Inner membrane was solubilized by resuspended the total membrane in PBS containing 0.01 mM MgCl_2_ and 2% Triton X-100 and at room temperature (RT) for 30 min. Subsequently, the outer membrane fraction was re-pelleted by centrifugation at 37,000 rpm for 4 h (at 4 °C), and the inner membrane remained in the supernatant. For purification of CarEW, *E. coli* BL21(DE3) cells containing pET-28a(+)/*carEW* plasmid were collected, disrupted and the supernatant was applied to a Ni^2+^-NTA agarose. Protein concentration was determined by the Bradford method using bovine serum albumin as standard [36].

For western blot analysis, cell-free extracts (crude extracts) and different cell fractions (~ 10 mg/ml of total protein of each sample) were separated by SDS-PAGE (12%), and then proteins were transferred (transfer buffer: 192 mM glycine, 25 mM Tris base, and 20% methanol, pH 8.0) onto a polyvinylidene difluoride (PVDF) membrane (Millipore, USA). Monoclonal His-tag antibody (IgG2), peroxidase-conjugated goat anti-mouse IgG (H + L) (both obtained from ZSGB-BIO, China), and a Super Signal West Pico kit from Thermo Scientific Pierce (USA) were used, and procedures were conducted following the method as reported by Nguyen et al. [26].

To investigate surface exposure of the CarEW, the proteinase K accessibility test was used. *E. coli* BL21(DE3) cells harboring pET-28a(+)/*inpn*/*carEW* (OD_600_ = 1.0) were incubated in PBS buffer (pH 7.0) with 100 μg/ml proteinase K (Sigma, USA) at 37 °C for 1 h, and the digest was terminated by adding 10 μM of phenylmethylsulfonylfluoride (PMSF) (Sigma, USA) following incubation on ice for 5 min. The proteinase K-treated and untreated cells were assayed for CarEW activity as described above.

### Fluorescence microscopic assay

After harvested and washed three times with PBS (pH 7.0), *E. coli* BL21(DE3) cells harboring pET-28a(+)/*inpn*/*carEW/gfp* plasmid *and* pET-28a(+)/*carEW/gfp* plasmid were diluted to OD_600_ 1.0, respectively. The green fluorescence was observed using LEICA DM6 B (Leica DMB, Wetzlar, Germany) with a 100-oil immersion objective and photographed with an N21 (BP 515-560) filter.

### Degradation of DiBP by cell surface-displayed CarEW

A 1-ml reaction mixture containing 10 U of surface-displayed CarEW or purified CarEW and 2 mg/ml DiBP (solubilized in 50 μl dimethyl sulfoxide (DMSO)) in 50 mM Tris/HCl (pH 9.0) was incubated at 45 °C. The reaction was terminated at indicated time points (0, 20, 40, 60, 80, 100, 120, 140, 160, 180 min) with 10% (v/v) 1 N HCl, and equal volume of ethyl acetate was used to extract reaction products. The extracts were re-dissolved in 1 ml of methanol after dried over Na_2_SO_4_, and the residual DiBP was analyzed on an Agilent gas chromatography-mass spectrometry (GC–MS) system (HP6890-5973 N).

The GC capillary column used was HP-5MS (0.25 mm by 0.25 μm by 30 m). The programmed oven temperature was as following: initial temperature of 60 °C for 1 min, followed by a 20 °C min^−1^ increase to 220 °C and maintained for 1 min; this was followed by a 5 °C min^−1^ increase to 250 °C and maintained for 1 min. This was followed by a 20 °C min^−1^ increase to 290 °C and was maintained for 7.5 min. The injector temperature was set to 260 °C and helium was used with a constant column flow rate of 1 ml min^−1^. One microliter of each sample extract was injected in splitless mode. Controls without enzyme were analyzed in parallel.

## Supplementary information

**Additional file 1: Figure S1.** Substrate specificity of *E. coli* BL21(DE3) displaying INPN-CarEW fusion protein towards acetylated esters with different lengths.

**Additional file 2: Figure S2.** Schematic diagram of the procedure to construct recombinant plasmids for bacterial cell surface display.

**Additional file 3: Table S1.** Plasmids and primers used in this study.

## Data Availability

All data generated or analyzed during this study are included in this published article and its additional files.
