# Supplementary figures and images for: Development of a whole-cell biocatalyst for diisobutyl phthalate degradation by functional display of a carboxylesterase on the surface of Escherichia coli
Source: Microb Cell Fact. 2020 May 29;19:114. doi: 10.1186/s12934-020-01373-6 (PMC7260753; doi:10.1186/s12934-020-01373-6)

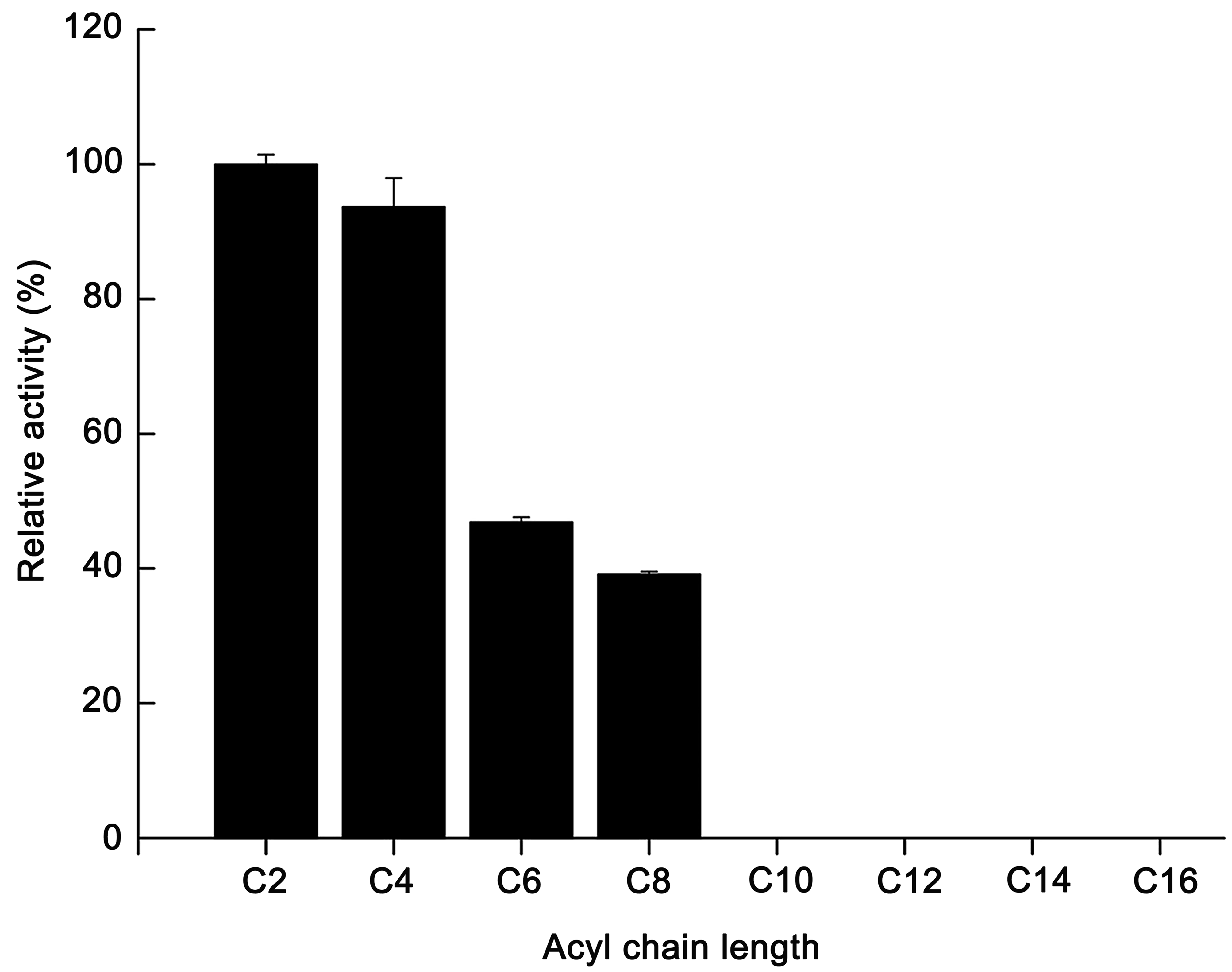

Supplement: Supplementary file 1 — Additional file 1: Figure S1. Substrate specificity of E. coli BL21(DE3) displaying INPN-CarEW fusion protein towards acetylated esters with different lengths. [file 12934_2020_1373_MOESM1_ESM.tif]

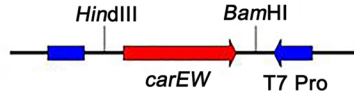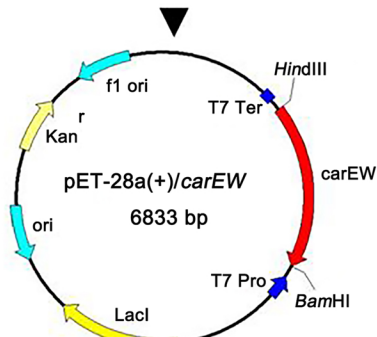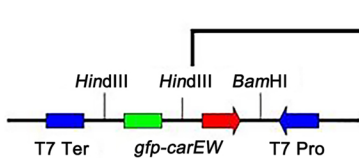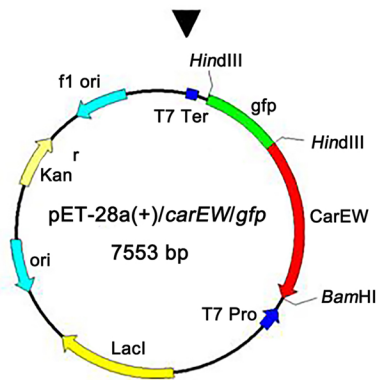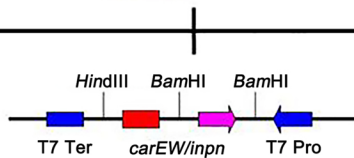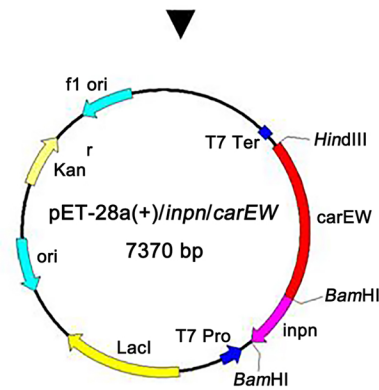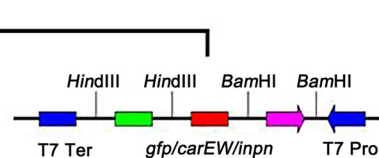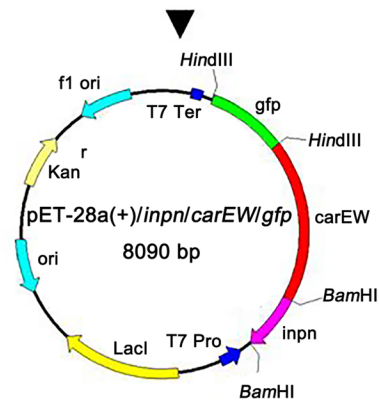

Supplement: Supplementary file 2 — Additional file 2: Figure S2. Schematic diagram of the procedure to construct recombinant plasmids for bacterial cell surface display. [file 12934_2020_1373_MOESM2_ESM.pdf]
