# Supplementary material for: Development of a whole-cell biocatalyst for diisobutyl phthalate degradation by functional display of a carboxylesterase on the surface of Escherichia coli
Source: Microb Cell Fact. 2020 May 29;19:114. doi: 10.1186/s12934-020-01373-6 (PMC7260753; doi:10.1186/s12934-020-01373-6)
Supplement: Supplementary file 3 — Additional file 3: Table S1. Plasmids and primers used in this study. [file 12934_2020_1373_MOESM3_ESM.doc]

**Table 1** Plasmids and primers used in this study

| Plasmids and primers | Description^a^ | Source or reference |
| --- | --- | --- |
| PlasmidspEGFP-N3pMD18-TpMD18-T/*inak* | Containing *gfp* gene (720 bp), Amp^r^T-clone vectorT-clone vector ligated with the synthesized *inak* gene (699 bp) from *Pseudomonas syringae*, Amp^r^ | TakaraTakara[21] |
| pEASY-E2/*carEW*pEASY-E2pET28a (+)pMD18-T/*inpn* | Expression vector coding for *carEW*Expression vectorExpression vectorContaining N-terminal of *inak* gene (537 bp), Amp^r^ | [9]TransGenNovagenThis study |
| pET28a (+)/*carEW* | Expression vector coding for *carEW*, Kan^r^ | This study |
| pET28a (+)/*carEW/gfp*pET28a (+)/*inpn/carEW*pET28a (+)/*inpn/carEW/gfp*Primers (5'→3') ^a^ | Expression vector coding for *carEW* and *gfp*, Kan^r^Expression vector coding for *carEW* and *inpn*, Kan^r^Expression vector coding for *carEW*, *inpn*, and *gfp*, Kan^r^ | This studyThis studyThis study |
| P1 | CGGGATCC ATGACTCATCAAATAGTAACG |  |
| P2P3 | CCCAAGCTT CTTCTCCTTTTGAAGGGAATAGCCCAAGCTT ATGGTGAGCAAGGGCGAGG |  |
| P4 | CCCAAGCTT CTTGTACAGCTCGTCCATGC |  |
| P5 | CG GGATCC ATGACTCTCGACAAGGCGTTG |  |
| P6P7P8 | CG GGATCC GGTCTGCAAATTCTGCGGCGTCCG GGATCC ATGGTGAGCAAGGGCGAGGCG GGATCC CTTGTACAGCTCGTCCATGC |  |

^a^ The restriction sites in the primers (5'→3') are underlined.
